# Progression in Fibrotic Interstitial Lung Diseases: Prevalence and Indicators in the Initial Evaluation in a Brazilian Multicentric Cohort

**DOI:** 10.7759/cureus.80290

**Published:** 2025-03-09

**Authors:** Ana C Resende, Soraya Cordero, Eliane V Mancuzo, Karin M Storrer, Maria A Moreira, Fernanda M Baptista, Rimarcs Ferreira, Maria Raquel Soares, Carlos Alberto C Pereira

**Affiliations:** 1 Department of Pulmonology, Federal University of São Paulo, São Paulo, BRA; 2 Department of Pulmonology, Federal University of Minas Gerais, Belo Horizonte, BRA; 3 Department of Pulmonology, Federal University of Paraná, Curitiba, BRA; 4 Department of Pulmonology, Federal University of Goiás, Goiânia, BRA; 5 Department of Pulmonology, São Rafael Hospital, Salvador, BRA; 6 Department of Pathology, Federal University of São Paulo, São Paulo, BRA

**Keywords:** chronic hypersensitivity pneumonitis, connective tissue disease, idiopathic pulmonary fibrosis, interstitial lung disease, prognosis, pulmonary progressive fibrosis

## Abstract

Objective: This retrospective study aimed to determine the prevalence of progression in fibrotic interstitial lung disease (ILD) and the findings at diagnosis most associated with progression after two years of follow-up in a large Brazilian cohort.

Methods: This was a retrospective multicenter observational study in Brazil. Progression was defined after two years of follow-up. We excluded patients with an initial peripheral oxygen saturation (SpO2) of less than 88% or an initial forced vital capacity (FVC) of less than 45%. Diagnoses were made by multidisciplinary discussion. Patients with idiopathic pulmonary fibrosis were included for comparison. At least one of the following events was indicative of progressive ILD: (1) a relative decrease in FVC of 10% or more, (2) worsening dyspnea, (3) a greater extent of fibrotic findings on high-resolution computed tomography (HRCT), (4) initiation of oxygen, and (5) death attributed to ILD. Logistic regression analysis was used to identify risk factors for progressive fibrosis.

Results: The mean age of patients was 61.7±12.3 years, and 69.5% had Velcro crackles. The mean FVC was 71.6±15.8%, and 26.1% showed honeycombing on HRCT. After two years of follow-up, 40.5% of patients (n=154) showed disease progression. Fibrotic hypersensitivity pneumonitis (FHP) was the most progressive disease (52%), and connective tissue disease-associated ILD (CTD-ILD) was the least progressive (25%). Multivariate analysis showed that a higher score for dyspnea, crackles, and SpO2 at rest ≤94% and ≤85% at the end of exercise were significant indicators of progression. Diffusing lung capacity for carbon monoxide (DLCO) was measured in 172 cases, with values <55% predicting a high odds ratio for progression (OR=4.03; 2.10-7.69).

Conclusion: In Brazil, FHP is the most progressive disease and CTD-ILD is the least progressive after two years of follow-up. The degree of dyspnea, crackles, SpO2 at rest and during exercise, and DLCO at baseline are associated with progressive disease.

## Introduction

Progressive fibrotic interstitial lung diseases (PF-ILDs) are a diverse group of conditions characterized by a similar course to idiopathic pulmonary fibrosis (IPF), with progressive loss of lung function and early mortality [1]. Irrespective of the underlying disease process, ILDs progress by similar mechanisms leading to fibroblast proliferation [2]. Recently, it has been suggested that these conditions be grouped under the term progressive pulmonary fibrosis (PPF) [3]. Progression can occur from months to years after diagnosis, but evaluation after one and two years are the most commonly used intervals [3,4].

Several endpoints, alone or in combination, can be used to assess disease progression [5]. Worsening symptoms, decline in forced vital capacity (FVC), reduction in diffusing lung capacity for carbon monoxide (DLCO), and increased fibrosis detected by high-resolution computed tomography (HRCT) are the most commonly used parameters [3,4].

In a prospective registry study, oxygenation status helped define disease progression in fibrotic ILD [6]. When patients with ILD require long-term oxygen therapy, their prognosis is poor [7]. Surprisingly, O2 desaturation or new-onset O2 use has not been included in the scores used to characterize progression in PPF. Ideally, indicators of progressive disease should be determined at the time of diagnosis to guide prognostic and therapeutic decisions. An expert group, after reviewing published work, suggested some risk factors for progression at initial assessment [8].

Dyspnea is the most important factor affecting the quality of life in people with IPF [9]. A recent study showed that greater baseline dyspnea remained significant as an indicator of progression on multivariate analysis in a group of patients with fibrotic ILD [10]. Honeycombing on HRCT is an indicator of poorer prognosis in diseases other than IPF [11]. However, there is only moderate agreement among chest radiologists, regardless of experience, regarding the presence of honeycombing on CT [12]. Velcro crackles on auscultation in ILD are associated with the pattern of usual interstitial pneumonia (UIP) or possible UIP on HRCT [13]. In one study, the presence of Velcro crackles predicted a worse prognosis in chronic hypersensitivity pneumonitis (HP) [14]. Pulmonary hypertension is known to complicate various forms of ILD and has been associated with worse outcomes [15].

The aim of the present study was to estimate the prevalence of progression of the most common fibrotic ILD in a retrospective multicenter Brazilian cohort and to identify baseline factors predictive of progression after two years of follow-up.

## Materials and methods

Study design and patients

This was a retrospective observational study of patients from six centers in Brazil: one private clinic and five university hospitals (Federal University of São Paulo, as the coordinating center, Federal University of Minas Gerais, Federal University of Paraná, Federal University of Goiás, and São Rafael Hospital).

The study was approved by the Research Ethics Committee of the Federal University of São Paulo (approval number: 6.627.739) and was also approved by a letter of consent from the other participating institutions. Informed patient consent was not required due to the retrospective design of the study.

Data collection

We collected data from electronic records from January 31, 2008, to January 31, 2020. Baseline data included age at diagnosis of ILD, gender, smoking history, presence of systemic findings, symptoms of gastroesophageal reflux disease (GERD), dyspnea score by Modified Medical Research Council (mMRC) [16], exposure assessment using a standardized questionnaire, family history of ILD, presence of Velcro crackles, pulmonary function tests (PFTs), and autoimmune serologies. These included anti-nuclear antibodies, rheumatoid factor, anti-cyclic citrullinated peptide (anti-CCP), extractable nuclear antigen antibodies, anti-Jo1, and, as of 2019, a comprehensive panel for anti-synthetase syndrome. Serum-precipitating antibodies for agents associated with HP are not available in Brazil. The presence or absence of honeycombing was based on the initial report from each center. PFTs performed at diagnosis and during follow-up included FVC and DLCO (as absolute and percentage of predicted value), O2 saturation at rest and at the end of exercise (four-minute step test or six-minute walk test), and transthoracic echocardiogram with estimated pulmonary systolic arterial pressure (ePSAP) by tricuspid regurgitation [17-19].

We also collected data on hospitalization, acute exacerbations attributed to ILD, and treatment (steroids and/or other immunosuppressants and antifibrotics).

Inclusion and exclusion criteria

Participants had to be at least 18 years of age and have evidence of fibrotic ILD (reticular abnormalities, traction bronchiectasis, honeycombing, or architectural distortion) on HRCT within six months of the initial visit. Patients were followed for at least two years.

Exclusion criteria were as follows: (1) fibrotic extent on HRCT <10% at baseline, (2) baseline FVC and at least one follow-up measure not available, (3) loss to follow-up during the first two years of the study, (4) an initial peripheral oxygen saturation (SpO2) ≤88%, (6) an initial FVC ≤45%, (7) exacerbation at the first visit, (8) presence of other potential causes of dyspnea, and (9) presence of fibrotic sarcoidosis and other conditions, such as a combination of pulmonary fibrosis and emphysema. Patients diagnosed with IPF were included for comparison.

Diagnostic criteria

Final diagnoses of ILD were made by local experts, including a pulmonologist, radiologist, and pathologist, based on clinical presentation, radiologic findings, and available histopathologic features, by multidisciplinary discussion (MDD). In case of disagreement with the primary center (n=21), the final diagnosis was done by a new MDD at the coordinating center. 

The diagnostic criteria were those suggested by specialized literature [20-26].

Progression criteria

At least one of the following events within two years was indicative of progressive ILD: (1) a relative decline in FVC of 10% or more; (2) worsening dyspnea in daily activities in comparison to the initial evaluation where patients with other causes of dyspnea during evolution, such as left heart failure, were excluded, but cases with pulmonary hypertension secondary to ILD were not; (3) a greater extent of findings indicative of fibrosis on HRCT; (4) initiation of oxygen; and (5) death ascribed to ILD. We excluded other causes of clinical or radiologic worsening. All of these outcomes were associated with shorter survival in this sample (data present in the American Thoracic Society (ATS) Conference 2023) [27].

Patients rated changes in dyspnea as better, worse, or unchanged from the first visit. Radiologists or experienced pulmonologists compared side-by-side images to assess fibrosis progression using HRCT. Each center defined the start of supplemental O2. Exacerbations and hospitalizations were not considered indicators of progression because of their unpredictability and association with other diseases in advanced stages.

Criteria progression was applied uniformly between centers and noted in electronic medical records for a period of at least two years from the initial evaluation. The progression criteria in this period were the last recorded. 

Statistical analyses 

Statistical analyses were performed using IBM SPSS Statistics for Windows, Version 25.0 (Released 2017; IBM Corp., Armonk, New York, United States). Summary statistics are presented as mean±SD or median (interquartile range) for continuous data and frequency (%) for categorical data. Baseline variables indicative of progression were selected by univariate and multivariate logistic regression analysis. Exercise SpO2 (ExSpO2), DLCO, and echocardiographically estimated pulmonary systolic arterial pressure (ePSAP) were not measured in all patients. Continuous variables were compared between progressive and non-progressive groups using Student's t-test or Mann-Whitney U test, and categorical variables were compared using Fisher's exact test or Pearson's chi-squared (χ2) test. For functional data, cut-off points between progressive and non-progressive cases were determined by receiver operating characteristic (ROC) curves using Youden's J index and the greater odds ratio around that point. Collinearity between variables was calculated by linear regression. The Kaplan-Meier survival analysis with the log-rank test was used to compare survival in patients with and without progressive disease. 

Survival was defined in months (from the date of ILD diagnosis) to the date of last assessment before loss to follow-up, death, or lung transplantation or until January 31, 2020. 

All-cause mortality was recorded. Statistical significance was set at p<0.05.

## Results

**Figure 1:**
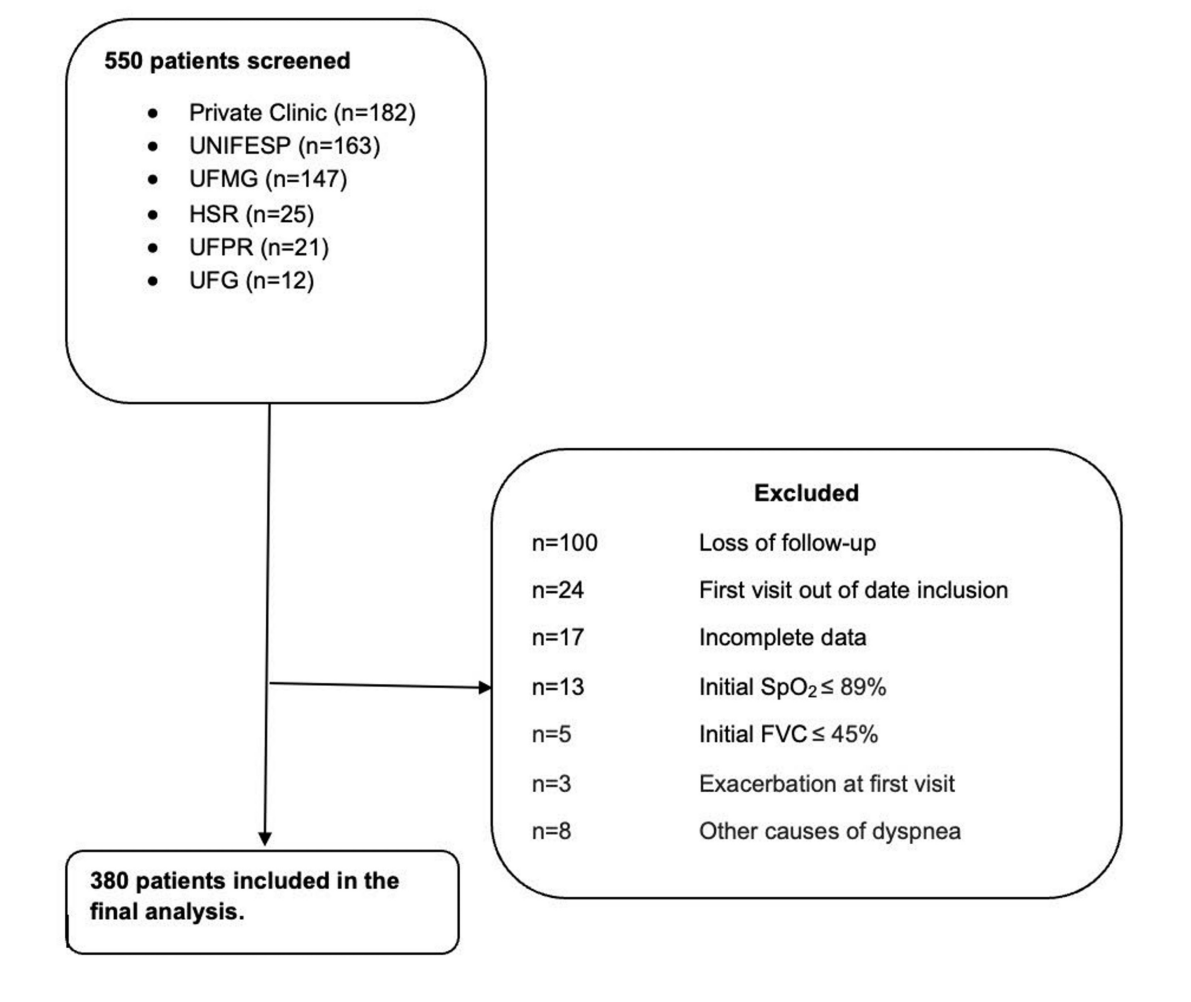
Flowchart showing the sample composition: patients screened from six reference centers, number of exclusions, and final number of patients included UNIFESP: Federal University of São Paulo; UFMG: Federal University of Minas Gerais; HSR: São Rafael Hospital; UFPR: Federal University of Paraná; UFG: Federal University of Goiás; SpO2: peripheral oxygen saturation; FVC: forced vital capacity

Initially, 550 patients were selected. Of these, 170 patients did not meet the study criteria and were excluded, leaving 380 in the final analysis (Figure 1).

After 24 months of follow-up, 53 (13.6%) patients had an exacerbation, and 62 (16.3%) patients required hospitalization. Antifibrotics were prescribed in 43 cases, 21 of which had IPF. One hundred and twenty-four (89%) cases with connective tissue disease (CTD) and 86 (80%) cases with fibrotic hypersensitivity pneumonitis (FHP) received immunomodulatory therapy. Patients treated with antifibrotic drugs had similar survival and rates of exacerbations in comparison to those not treated (data not shown).

After two years of follow-up, 154 (40.5%) had progressive disease. Variables indicating progression in decreasing order were a decrease in FVC (n=122), worsening of fibrosis on HRCT (n=117), worsening of dyspnea (n=109), and requirement of supplemental oxygen (n=61). In 10 of these cases, dyspnea was the only progression criterion present. The odds ratio for mortality for those with worsening dyspnea was 4.11 (95% CI=2.68-6.32; p<0.001).

In a specific analysis of our cases with long-term oxygen therapy (data not shown), the factors associated with new-onset oxygen therapy, by multivariate analysis, were lower SpO2 at exercise, a higher grade of dyspnea, an ILD diagnosis other than CTD, and a lower FVC% predicted. The most significant variable in the multivariable logistic regression was SpO2 ≤85% at exercise. The odds ratio for mortality for O2 use was 7.09 (95% CI=4.58-10.98; p<0.001).

Table 1 shows the number of cases and the percentage of progressors based on the final diagnosis. Except for ILD-CTD, which progressed in 35 (25%) cases, other diseases showed progression in more than 45% of cases.

**Table 1:** Diagnosis according to progression ILD: interstitial lung diseases; FHP: fibrotic hypersensitivity pneumonitis; IPF: idiopathic pulmonary fibrosis; CTD: connective tissue disease; IPAF: interstitial pneumonia with autoimmune features *Others: idiopathic nonspecific interstitial pneumonia (8), idiopathic bronchiolocentric interstitial pneumonia (1), fibrosis ascribed to microaspiration (3), and familial interstitial fibrosis (3)

ILD groups, total number	Progressive, number (%)
FHP, n=107	56 (52.3%)
IPF, n=81	38 (46.9%)
Not classified, n=38	18 (47.4%)
Others, n=15*	7 (46.7%)
CTD, n=139	35 (25.2%)
Systemic sclerosis, n=62	16 (25.8%)
Rheumatoid arthritis, n=29	4 (13.7%)
IPAF, n=20	10 (50%)
Mixed CTD, n=9	2 (22.2%)
Myositis, n=11	2 (18.2%)
Sjögren's syndrome, n=8	1 (12.5%)

**Figure 2:**
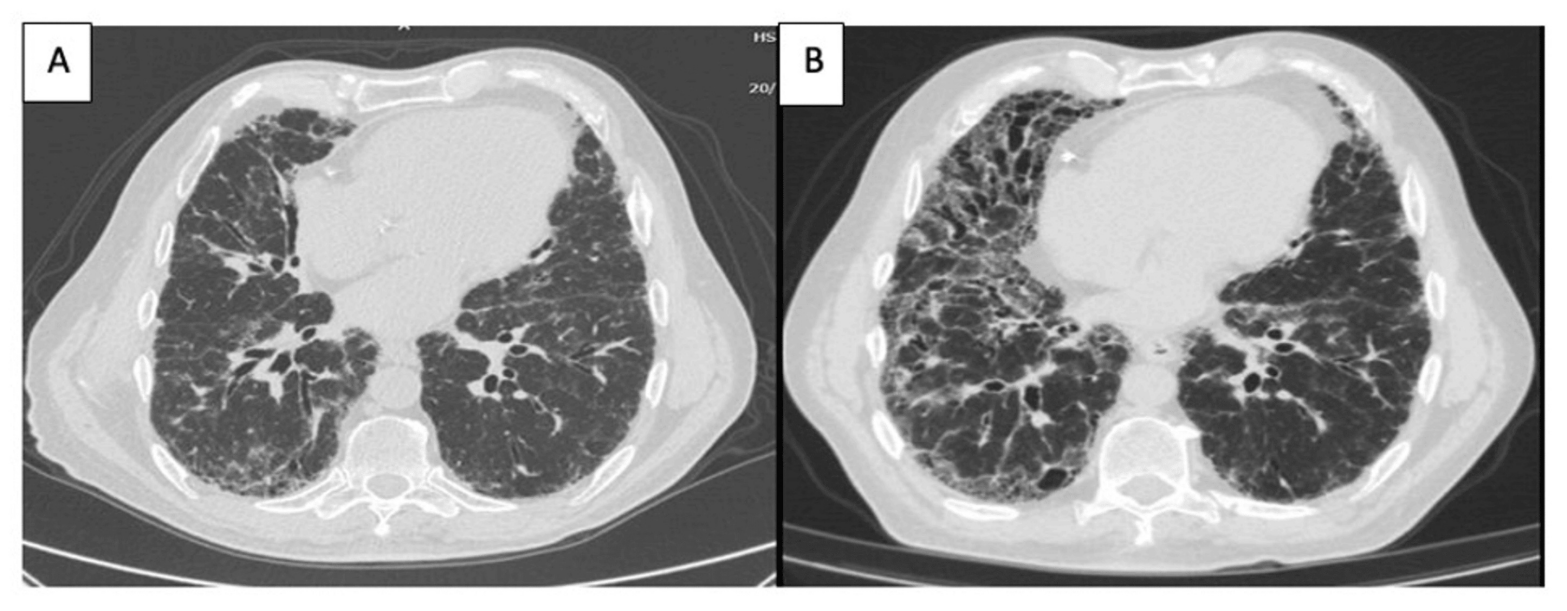
HRCT of a case with HP: (A) image during the initial evaluation and (B) after two years of follow-up. The images show a significant progression of the fibrotic pattern, increased ground glass, and increased traction bronchiectasis HRCT: high-resolution computed tomography; HP: hypersensitivity pneumonitis

An HRCT of a patient with FHP at baseline is shown in Figure 2A and after two years of follow-up in Figure 2B.

Females predominated in CTD. Systemic sclerosis (SS) and rheumatoid arthritis (RA) were the most common CTDs. ILD associated with RA had a low rate of progression (14%). In nine (31%) RA cases, HRCT showed the UIP pattern. ILD was treated in 49 (79%) cases with ILD-SS and in 27 (92%) cases with ILD-RA.

Table 2 shows the baseline characteristics of the entire sample and compares progressors and non-progressors. The mean age of the patients was 61.7 years, and 212 (55.8%) were female. On lung auscultation, 264 (69.5%) of the patients had Velcro crackles, and 97 (25.5%) had honeycombing on HRCT. Two hundred and seventeen (57.1%) cases had potential exposure for HP. Of these, only 107 (49.3%) had a final diagnosis of FHP. Otherwise, all patients with a final diagnosis of FHP had potential exposure. The mean FVC was 71.6%. Only 172 patients had DLCO measured, with a mean of 56.8%, and 269 patients had ExSpO2 measured, with a mean of 87.4%. 

**Table 2:** General data of patients with fibrotic interstitial lung disease, separated by progressive or non-progressive course GERD: gastroesophageal reflux disease; CTD: connective tissue disease; FVC: forced vital capacity; DLCO: diffusing lung capacity for carbon monoxide; SpO2: peripheral oxygen saturation; ePASP: estimated pulmonary artery systolic pressure Statistical test used to calculate the p-values: *: t-test, **: χ2, and ***: median test

Variables	All patients, n=380	Progressive, n=154	Non-progressive, n=226	P-value	Test
Age, ×±SD (years)	61.7±12.3	63.1±11.1	60.8±13.1	0.08	1.78*
Male, n (%)	168 (44.2%)	79 (51.3%)	89 (39.4%)	0.02	5.27**
Smoker: past or current, n (%)	169 (44.5%)	74 (48.1%)	95 (42%)	0.25	1.34**
Family history, n (%)	61 (17.2%)	23 (15.5%)	38 (18.4%)	0.62	0.24**
GERD symptoms	204 (53.7%)	85 (55.5%)	119 (52.7%)	0.62	0.24**
Dyspnea, score, MD, IQR	1 (1-2)	2 (1-2)	1 (1-2)	0.03	Median test***
Velcro crackles, n (%)	264 (69.5%)	123 (79.9%)	141 (62.4%)	<0.01	13.19**
Honeycombing on HRCT, n (%)	99 (26.1%)	47 (30.5%)	52 (23%)	0.10	2.78**
CTD, n (%)	139 (36.6%)	27 (19.4%)	112 (80.6%)	<0.01	40.49**
FVC (%), ×±SD	71.6±15.8	67.7±14.5	73.3±16.4	<0.01	3.42*
DLCO (%), ×±SD (n=172)	56.8±15.8	51.0±16.3	60.6±14.3	<0.01	2.88*
Rest SpO2 (%), ×±SD	94.9±2.12	94.4±2.1	95.3±12.1	<0.01	4.10*
SpO2 at exercise(%), ×±SD (n=269)	87.4±5.9	85.5±6.2	88.8±5.4	<0.01	4.56*
ePASP (n=124)	35.0±12.3	39.9±15.0	30.4±6.4	<0.01	4.31*

Univariate logistic regression revealed several distinct baseline factors when comparing progressors and non-progressors (Table 3).

**Table 3:** Variables indicative of progression in fibrotic interstitial lung disease, selected by logistic univariate analysis in the initial evaluation (n=380) CTD: connective tissue disease; SpO2: peripheral oxygen saturation; mMRC: Modified Medical Research Council; FVC: forced vital capacity; DLCO: diffusing lung capacity for carbon monoxide; HP: hypersensitivity pneumonitis; ePASP: estimated pulmonary artery systolic pressure; OR: odds ratio; 95% CI: 95% confidence interval Statistical test used to calculate the p-values: univariate logistic regression

Indicators of progression	OR, 95% CI	P-value
Age	1.02 (0.99-1.03)	0.08
Male	1.62 (1.07-2.45)	0.02
Rest SpO2 ≤94%	2.39 (1.48-3.86)	<0.01
Baseline dyspnea (mMRC ≥2)	1.76 (1.6-2.66)	<0.01
Velcro crackles	2.39 (1.48-3.86)	<0.01
Honeycombing on HRCT	1.47 (0.92-2.33)	0.10
CTD	0.36 (0.22-0.58)	<0.01
FVC, %	0.98 (0.96-0.99)	<0.01
FVC <65%	1.59 (1.04-2.43)	0.03
DLCO (%), (n=172)	0.96 (0.94-0.98)	0.01
DLCO <55%	4.03 (2.10-7.69)	<0.01
Peak exercise SpO2 (%), (n=269)	0.91 (0.87-0.95)	<0.01
Exercise SpO2 ≤85%	3.52 (2.03-6.10)	<0.01
ePASP (n=124)	1.11 (1.05-1.16)	<0.01
ePASP >40 mmHg	5.10 (1.97-13.0)	<0.01

Progressors were more likely to be male and slightly older. They had more exposure to HP-related antigens, more Velcro crackles, a higher dyspnea score, fewer diagnoses of CTDs, and worse lung function, especially in terms of gas exchange parameters.

In univariate analysis, the presence of honeycombing on HRCT was marginally associated with progression (p=0.10). The presence of Velcro crackles was associated with honeycombing on HRCT. Of 99 patients with honeycombing, 91 (92%) had Velcro crackles on auscultation compared to 173 of 281 (62%) without honeycombing (χ2=31.8; p<0.01).

ExSpO2, DLCO, and estimated pulmonary artery systolic pressure (ePASP) in echocardiogram were measured in smaller numbers of patients, but all showed significant associations with the likelihood of disease progression. The best cut-off points to discriminate between those who progressed and those who did not were DLCO <55%, ExSpO2 ≤85%, resting SpO2 ≤94%, FVC <65% predicted, and ePASP >40 mmHg. 

Multivariable analysis of all 380 cases showed that shortness of breath (mMRC ≥2), Velcro crackles, and resting SpO2 ≤94% predicted a higher risk of progression. We repeated the analysis by adding ExSpO2 (n=269) and resting SpO2 (variables not collinear; variance inflation factor=1). ExSpO2 was included in the model, but not rest SpO2 or dyspnea (Table 4).

**Table 4:** Variables indicative of progression in fibrotic interstitial lung disease, selected by logistic multivariate analysis in the initial evaluation (n=380) CTD: connective tissue disease; SpO2: peripheral oxygen saturation; HP: hypersensitivity pneumonitis; ePASP: estimated pulmonary artery systolic pressure; OR: odds ratio; 95% CI: 95% confidence interval *When multivariate analysis was repeated introducing SpO2 at exercise (n=269), excluding SpO2 at rest, OR was 3.74, and 95% CI was 2.09-6.71. Dyspnea was excluded from the model. Statistical test used to calculate the p-values: multivariate logistic regression

Indicators of progression		OR, 95% CI		P-value
Rest SpO2 ≤94%		1.97 (1.26-3.08)		<0.01
Baseline dyspnea (mMRC ≥2)		1.73 (1.10-2.71)		0.02
Velcro crackles		2.20 (1.33-3.65)		<0.01
CTD		0.32 (0.20-0.52)		<0.01
Exercise SpO2 ≤85%		*3.74 (2.09-6.71)		<0.01

**Figure 3:**
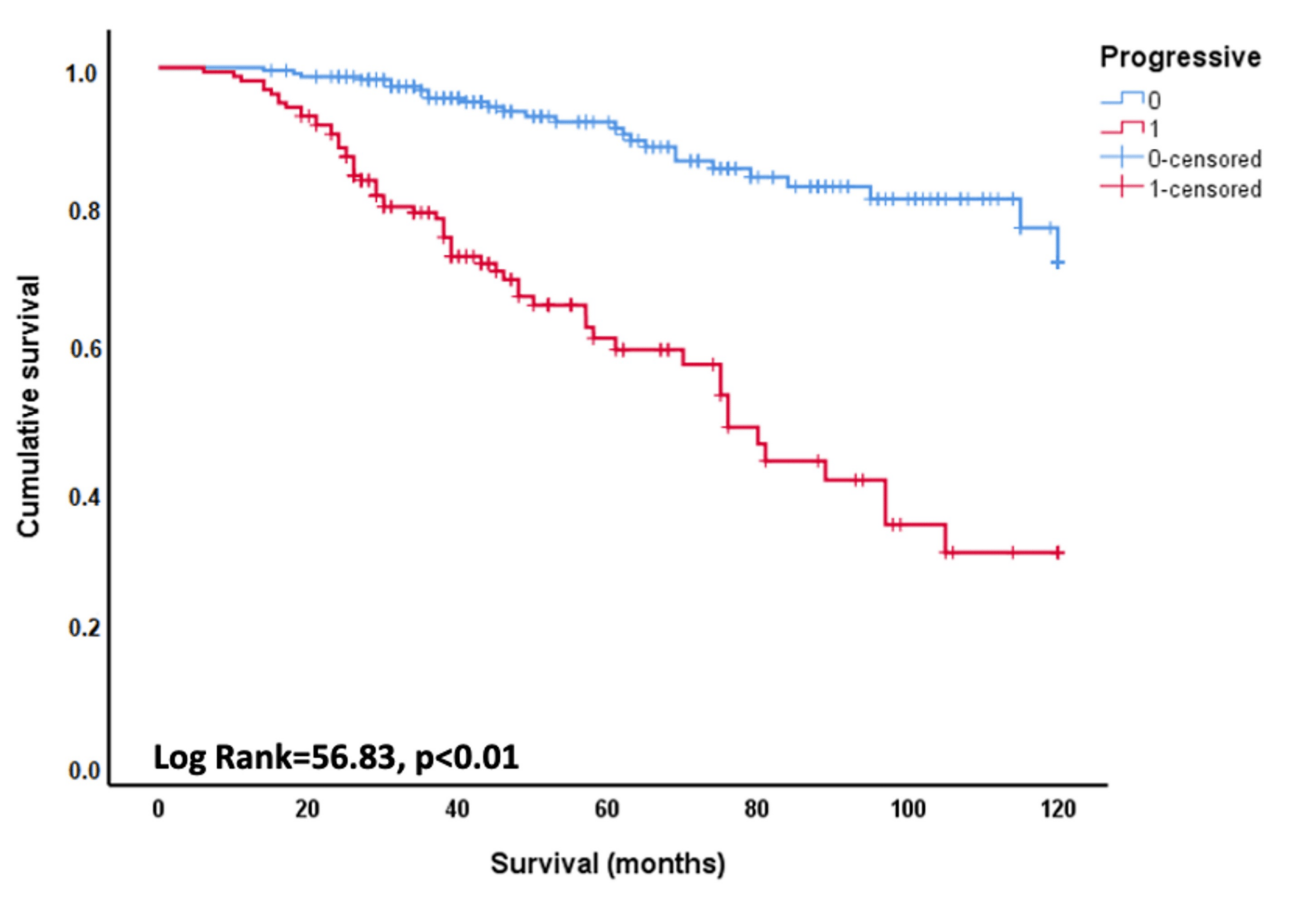
Survival in patients with fibrotic interstitial lung disease, progressors and non-progressors after two years of follow-up

The median follow-up for the entire sample was 45.5 (IQR=31.0-76.0) months. Survival was longer in non-progressive patients (log-rank=56.83; p<0.001) (Figure 3). The median survival was 74.4 months (95% CI=66.6-82.3) for progressors and indeterminate for non-progressors. At five years, mortality was 40% in progressors compared to 9% in non-progressors. When the analysis was repeated, excluding 81 patients with IPF, we found a very similar result: the five-year mortality was 37% in progressive cases compared with 9% in non-progressive cases.

## Discussion

This Brazilian multicenter retrospective study investigated the risk factors associated with the progression of the most common fibrotic ILDs over time. Within 24 months, approximately 40% of patients showed progression as defined by clinical, radiographic, and physiological criteria, with the highest rates observed in HP (52%) and IPF (47%) and the lowest in CTD-ILD (25%).

In our view, progression criteria for ILD are a topic still in progress. In this cohort, we found that each variable selected as a progression criterion correlated with survival (data present in the ATS Conference 2023) [27].

In a large multicenter study (n=2746) of patients with fibrotic ILD (including IPF), 50% had PPF 24 months after diagnosis. PF-ILD occurred in 427 (59%) patients with IPF and 125 (58%) with fibrotic HP [28].

The definition of PPF excludes patients with IPF. However, similar to other studies, we included IPF for comparison with the non-IPF cohort [26,28]. IPF is widely accepted as the prototypical PF-ILD. Some studies estimate progression rates in IPF to be as high as 95%, but others report lower percentages [28]. In Brazil, many cases with an HRCT pattern of "probable IPF" have FHP on surgical lung biopsy, making differential diagnosis difficult [29].

The UIP pattern with honeycombing has been associated with poorer prognosis in several ILDs. In our study, honeycombing was a relatively poor predictor of progression. This has been shown by others [30].

Irrespective of the inclusion of IPF, the present study clearly associated the progressive course of fibrotic ILD with survival. This has also been shown in other studies [1].

In the present study, patients with CTD-ILD had a significantly lower risk of progression compared to other diagnoses, so differentiation from other diseases at the initial evaluation is critical. A very large retrospective cohort showed that initial progression was significantly less rapid in patients with CTD-ILD, particularly in those with inflammatory myopathy [31]. The use of immunosuppressants is a potential cause of the observed outcomes due to the inflammatory nature of these diseases [32].

A previously published study by our group demonstrated that patients with HP, even fibrotic ones, can remain stable without pharmacological treatment as long as the antigen is removed [33].

We do believe that treatment with antifibrotics is an important advance in fibrotic ILD, but in Brazil, a developing country, access to antifibrotics is very complicated. Due to the high cost, the medications have not been released for prescription by the universal health system, to which everyone is entitled (Sistema Único de Saúde (SUS) or Unified Health System), and are also not included and allowed for prescription by private health plans. Patients must resort to the courts to receive the medication, which restricts its use. This persists to this day.

Multivariate analysis showed that dyspnea, crackles, SpO2 at rest ≤94%, and ExSpO2 ≤85% were associated with a higher risk of progression. Similar to mortality studies in IPF, we found that dyspnea had a significant and independent role in predicting progression in PPF [34].

As shown in the "Data collection", dyspnea was scored in the initial evaluation by mMRC [16], and changes were rated as better, worse, or unchanged from the first visit, considering daily activities. Baseline and longitudinal changes in dyspnea are significant predictors of outcomes in chronic ILD, including both IPF and non-IPF ILD [35,36].

Larger studies in IPF have proposed several cut-off points, with lower FVC% values indicating progressively higher mortality risk [37]. In our study, a cut-off of 65% for FVC% was found to be the best discriminator, but this threshold was not selected for multivariate analysis. This cut-off is similar to that found in a large study of IPF after one year of follow-up [38].

It should be noted that SpO2 at rest, which is a very simple measure, indicated a greater risk of progression when it was ≤94% and this finding remained significant in the multivariate analysis. Unfortunately, not all cases in the present study had measurements of DLCO and ExSpO2. ExSpO2, which was measured in 71% of the sample, showed that for values ≤85%, odds ratios for progression greater than 3.5 were observed in both univariate and multivariate analyses. This cut-off is similar to that found in another study of 173 patients with IPF from our group, but it is lower than the cut-off of 88% during the six-minute walk test proposed in a classic study published on this topic [34,39].

The indication of O2 for ILD in literature is not clear; sometimes, it is based on criteria suggested for chronic obstructive pulmonary disease (COPD). As shown by others, oxygenation status provides prognostic information in PPF and may assist in defining disease progression in fibrotic ILD [40].

DLCO is the functional variable that best correlates with disease extent in IPF and is also the variable that most reliably predicts survival at baseline [41,42]. In 172 cases where DLCO was measured, we found an odds ratio of 4.03 in univariate analysis.

We found that the presence of Velcro crackles, an easily identified sign, was a risk factor for disease progression in both univariate and multivariate analyses of fibrotic ILD. We also found an association between Velcro crackles and honeycombing on HRCT, but crackles were a better predictor of progression. 

Several limitations should be recognized. Retrospective studies from tertiary centers may introduce selection bias. Lack of follow-up resulted in the exclusion of many patients. Relevant criteria such as acute exacerbations of ILD, hospitalization, and ILD-related death in the first two years were not included as progression criteria because no uniform criteria were used to characterize exacerbations. Although the definition of PF-ILD used in the study was not a consensus definition, we do not believe that this influenced the results. Two years of observation to characterize progression seems to be a good compromise [43]. Local preferences dictated the treatment of patients, making it impossible to disentangle the effect of the indication on outcomes, but the treatment indications were all carried out in reference centers with great experience in ILD. Finally, it is becoming clear that the extent of fibrosis on HRCT, as determined by quantitative CT analysis, is a key factor in PPF but was not available during the study period [44].

## Conclusions

The present study provides information on a clinical cohort of patients with PF-ILDs in a real-world setting in a developing country. FHP patients accounted for the majority of progressors during 24 months of follow-up, and ILD-CTD was the least progressive disease.

Smaller rest and exercise SpO2 levels and lower DLCO are linked to a higher likelihood of progression in patients with PF-ILDs at the time of initial examination. Dyspnea and Velcro crackles were also associated with a greater risk of progression. These parameters are widely available in the initial clinical evaluation. In general, the progressive course of the disease is associated with a poor prognosis. 
